# Increasing Seriousness of Plant Invasions in Croplands of Eastern China in Relation to Changing Farming Practices: A Case Study

**DOI:** 10.1371/journal.pone.0074136

**Published:** 2013-09-16

**Authors:** Guo-Qi Chen, Yun-He He, Sheng Qiang

**Affiliations:** 1 Weed Research Laboratory, Nanjing Agricultural University; Nanjing, China; 2 Department of Landscape Architecture, Zhejiang Agricultural and Forestry University, Hangzhou, China; Beijing Forestry University, China

## Abstract

Arable areas are commonly susceptible to alien plant invasion because they experience dramatic environmental influences and intense anthropogenic activity. However, the limited reports on relevant factors in plant invasion of croplands have addressed single or a few invasive species and environmental factors. To elucidate key factors affecting plant invasions in croplands, we analyzed the relationship between 11 effective factors and changes in composition of alien plants, using field surveys of crop fields in Anhui Province conducted during 1987–1990 (historical dataset) and 2005–2010 (recent dataset), when rapid urbanization was occurring in China. We found that in the past few decades, the dominance and richness of alien plant populations approximately doubled, despite differences among the 4 regions of Anhui Province. Among the 38 alien invasive plant species observed in the sites, the dominance values of 11 species increased significantly, while the dominance of 4 species decreased significantly. The quantity of chemical fertilizer and herbicide applied, population density, agricultural machinery use, traffic frequency, and annual mean temperature were significantly related to increased richness and annual dominance values of alien plant species. Our findings suggest that the increase in alien plant invasions during the past few decades is primarily a result of increased application of chemical fertilizer and herbicides.

## Introduction

With their high levels of available resources and anthropogenic disturbance, agricultural areas are particularly susceptible to alien plant invasions [1], [2].The continuous introduction and expansion of invasive plants into arable areas makes the management of alien crop weeds increasingly challenging. It is critically important to measure the seriousness and potential trends of alien weed invasions in arable lands, in order to form strategies for the management of these species [3].

Invasive plant species are generally capable of rapid adaptation to altered environments, climate, and human disturbance [1], [2], and shifts in farming practices may facilitate plant invasions in arable areas [4]. For example, overuse of herbicides may lead to outbreaks of resistant or tolerant invasive weed populations; 346 herbicide-resistant biotypes of 194 weed species have been described [5], of which many (e.g., *Lolium multiflorum*, *Sorghum halepense*, and *Conyza canadensis*) are highly invasive. Long-term application of chemical fertilizer may promote alien plant invasions in agricultural areas, as habitats with higher levels of nutrient resources tend to be more susceptible to plant invasions [6], [7]. Kovacs-Hostyanszki et al. [8] found that fertilizer had a negative impact on the richness of weed species with lower nitrogen preference, and on the coverage of native weeds. In addition, use of agricultural machinery may disperse seeds or other propagules (e.g., rhizomes) of invasive plant species over great distances [9], [10]. Climate change also facilitates range expansion of many invasive plant species [3], such as *Sorghum halepense* [11] and *Carduus nutans* [12]. Comparative studies examining the relative influence of the wide variety of factors involved in plant invasion in arable areas are scarce.

In China, large numbers of invasive plant species have established and spread rapidly in arable areas since the 1980s [13]. Changes in the dynamics of plant invasions have occurred concomitantly with changes in agrochemical inputs, land use [13], farming methods [14], and climate [15]. Before 1980, weed control practices in China were limited to manual removal, tillage, and adjustments in crop rotation. Herbicide application began during the 1980s, and expanded rapidly to become the primary weed management strategy after the middle 1990s [14]. In addition, rapid urbanization was accompanied by abandonment of rural areas and farming by large numbers of people, leading to increased mechanization and fertilizer use. The effects of climate change have also become more apparent since the 1980s [15]. The combination of these diverse changes in environmental factors and farming practices may lead to profound changes in plant community dynamics. However, few studies have addressed the invasion of crops by alien weeds in China. In this study, we assessed weed species richness and dominance in croplands in Anhui Province, a typical agricultural province in eastern China. We hypothesized that increasing applications of chemical herbicide and fertilizer, traffic frequency, and population density may promote alien plant invasions in croplands. Climate change may also have a strong influence on alien weed invasions in cropland. To test this hypothesis, we analyzed 2 datasets obtained from field surveys of summer crops (wheat and oilseed rape) conducted during 1987–1990and 2005–2010, and explored the key factors responsible for changes in alien plant species richness and dominance.

## Materials and Methods

### Ethics Statement

Here, by conducting field surveys, we studied weed communities in croplands in Anhui Province, China. No specific permissions were required and the field studies did not involve endangered or protected species.

### Study Area

**Figure 1:**
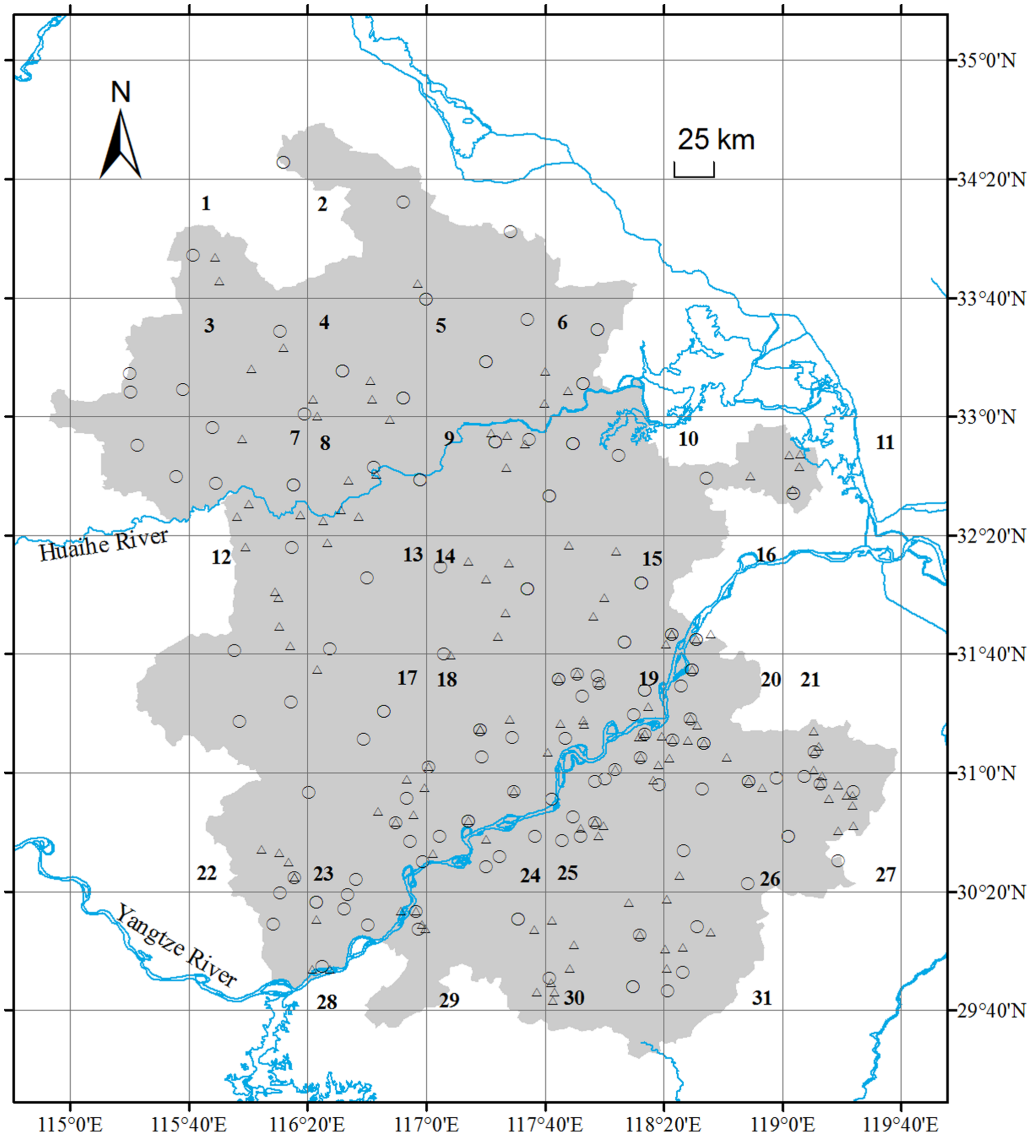
Sites surveyed during 1987–1990 (Δ) and 2005–2010 (○) in summer crop fields in Anhui Province, China.

Anhui Province (29°24′ to 34°57′N lat, 114°53′ to 119°39′E long) is located in eastern China with a total area of approximately 1.4×10^5^ km^2^ (Fig. 1). The province differs in climate from north to south. Average annual temperature ranges from 14 to 17°C, average rainfall from 800 to 1800 mm y^−1^, and average frost-free period from 200 to 250 d [16].

### Data Collection

Two datasets from field surveys made approximately 15 years apart in Anhui province (Fig. 1) were used to estimate changes in weed species richness and dominance. Field surveys were conducted from 1987 to 1990 (“historical dataset,” by Sheng Qiang [17]) and from 2005 to 2010 (“recent dataset,” this study, by Guo-Qi Chen and Yun-He He) using the same methods. All surveys were conducted after crops (wheat, *Triticum aestvum* L. or oilseed rape, *Brassica napus* L.) flowered, but before harvest. Eighty-three sites cultivated with oilseed rape and 47 sites with wheat were surveyed from 1987 to 1990, and 78 sites cultivated with oilseed rape and 69 sites with wheat were surveyed between 2005 and 2010. In Anhui Province, the amount of arable land per capita is approximately 0.06 ha [16], and most croplands are divided into small units with clear margins. At each survey site, we established ten 0.06-ha quadrats (approximately 666 m^2^) [17] in which we recorded all weed species and their dominance values. Dominance values were divided into 7 categories according to relative coverage, abundance, and height of each weed species (Table 1). This method is one of the most common protocols employed in arable weed field surveys in China [17].

**Table 1:** Visual scoring method for weed dominance value in crop fields.

		Maximum height in field	
Code	>80 cm	20cm–80 cm	<20cm
0.1	1–3 stems or total coverage <0.1%	<10 stems or total coverage <1%	<15 stems or total coverage <2%
0.5	4–10 stems or total coverage 0.2%–0.9%	11–15 stems or total coverage 1%–2%	16–30 stems or total coverage 3%–5%
1	11–15 stems or total coverage 1%–2%	16–30 stems or total coverage 3%–5%	31–60 stems or total coverage 6%–10%
2	16–30 stems or total coverage 3%–5%	31–60 stems or total coverage 6%–10%	61–100 stems or total coverage 11%–25%
3	31–60 stems or total coverage 6%–10%	61–100 stems or total coverage 11%–25%	101–200 stems or total coverage 25%–50%
4	61–100 stems or total coverage 11%–25%	101–200 stems or total coverage 25%–50%	201–500 stems or total coverage 50%–90%
5	>100 stems or total coverage >25%	>200 stems or total coverage >50%	>500 stems or total coverage >90%

In order to determine the key factors affecting the increasing dominance and richness of invasive plant species in the surveyed summer croplands, we analyzed 11 environmental factors relating to crop type, crop rotation, climate, and human disturbance and farming mode (Table 2). We obtained these data for each site and survey year from records in the Anhui Statistical Yearbook [16]for the cities in which field sites were located. We obtained data on herbicide application from the Yearbooks and from local Plant Protection Stations, which were government organizations, which were responsible for consisting farmers cultivating crops, as well as introducing pesticides to farmers. Two types of crop rotations were employed in the surveyed croplands: (1) wet-dry rotation consisting of rice followed by wheat (oilseed rape); and (2) dry crop rotation, which was just2 dry crops in succession, such as corn (soybean) –wheat (oilseed rape) rotation.

**Table 2:** Mean values of environmental factors of 130 historical sites and 147 recent sites examined in this study.

Factor	Historic	Recent
Crop type (oilseed rape or wheat)	–	–
Crop rotation (wet-dry crop or just dry crops) ^A^	–	–
Mean temperature of the coldest month (January, °C)	3.79	3.09*
Mean temperature of the hottest month (July, °C)	28.45	28.52 *^NS^*
Annual mean temperature (°C)	15.88	16.47*
Annual mean precipitation (mm)	1671.02	1239.29*
Population density (people/km^2^)	329.54	485.30*
Traffic frequency (Freight turnover (10^4^ ton km/km^2^))	7.02	341.74*
Net cropland agricultural machinery power (kw/ha.)	4.01	12.11*
Net cropland chemical fertilizer applied (kg/ha.)	374.66	782.20*
Net cropland herbicide applied (kg/ha.)	0.53	9.77*

For each environmental factor, mean values of historical and recent datasets in 31 grids (see Figure 1) were compared with paired-sample t-tests. Note: ^A^: the preceding crops in fields with wet-dry crop rotation were wet rice, while those in lands with dry crop rotation were dry crops such as soybean, corn, and cotton. “*”: *P*<0.05 and “*^NS^*”: not significant.

### Data Analysis

Data matrices for the historical and recent datasets included alien weed richness and dominance value and environmental factors for each site. Mean dominance values by species were calculated for each site from the 10 quadrats to obtain a site-species data matrix. Shannon-Weiner α-diversity [18] was then calculated for both datasets. For each site, the number of alien weed species was estimated as the richness value for each site, and the sum of the dominance values of all alien weed species was calculated as overall dominance value.

To better test the relationship between changes in alien weed invasions and changes in environmental factors, we divided the surveyed area (Anhui Province) into small grids, each of which covered a geographic area of 40′×40′. The surveyed area included31 grids that contained both the historical and recent survey sites (see Fig. 1), which were used as the sample units for the analyses. For each index in a given grid, we used the average value of the sites located in that grid. We then calculated the change in each index for a given grid by the value in the recent dataset minus that for the related historical data. For each environmental factor, mean values of historical and recent datasets in the 31 grids were compared using paired-sample t-tests. On the basis of the data from these grids, we employed redundancy analysis (RDA) to test the relationship between changes in environmental factors and changes in dominance values of alien weed species, using the “vegan” add-on package in the R 2.12.1 Language and Environment for Statistical Computing [19].

Stepwise regression models [20], [21] were used to explore relationships between changes in environmental factors and changes in total richness and dominance of alien weeds. The best-fit models were selected using Akaike’s information criterion (AIC) [22], [23]. Changes in total richness and dominance values, amount of herbicide and chemical fertilizer applied, and agricultural machinery power were calculated as ratios of recent to historical data. Changes in precipitation and temperature were calculated by subtracting historical values from recent values.

## Results

Human disturbance factors and mean annual temperature increased significantly (*P*<0.05) from 1987 to 2010, while mean temperature of the coldest month and annual precipitation decreased significantly (Table 2). Compared with the historical dataset, the amount of chemical fertilizer applied doubled, the amount of chemical herbicide applied increased by 18 times, and traffic frequency was 49 times greater in the recent dataset. Moreover, both the overall richness and dominance values of alien weeds doubled.

**Figure 2:**
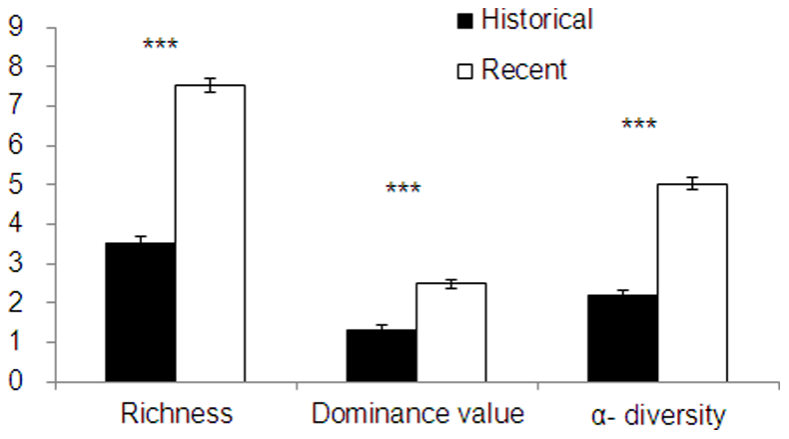
Comparisons between the historic and recent datasets in richness (number of species per site), dominance value, and α-diversity of overall alien weed species in summer crop fields in Anhui Province, China. Note: “***”: *P*<0.001.

**Figure 3:**
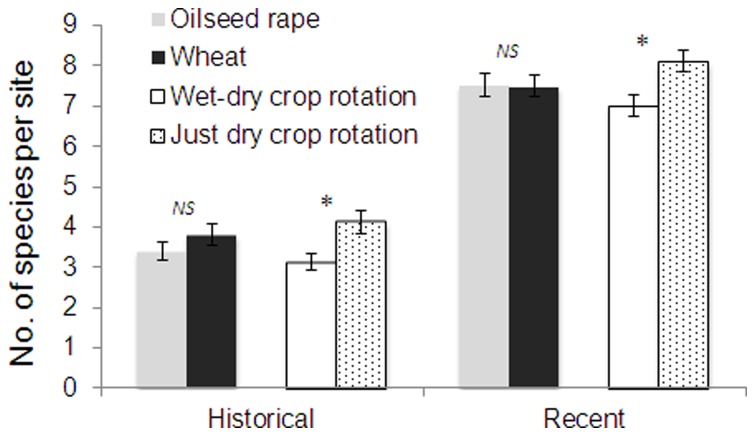
Overall dominance values of alien crop weeds in different groups of summer croplands surveyed in Anhui Province, China.

In the historical dataset, 24 alien weed species from 17 genera and 10 families were recorded (Table 3). Among these, 9 species (37.5%) had frequencies higher than 10% and 2 (8.3%) had frequencies higher than 50%. The average number of alien weed species per site was 3.65 (Fig. 2). In the recent dataset, 35 alien weed species from 26 genera and 13 families were recorded (Table 3). Among these, 17 (48.57%) had a frequency higher than 10% and 6 (17.14%) had a frequency higher than 60%.Overall richness, dominance value, and α-diversity of alien weeds in the recent dataset were significantly higher (*P*<0.001) than the relative values in the historical dataset (Fig. 2). Wheat fields had a significantly higher (*P*<0.01) occurrence of alien weeds than oilseed rape fields in the historical dataset, but not in the recent dataset (Fig. 3). Croplands with wet-dry crop rotation (rice as the preceding crop) showed significantly lower dominance of alien weeds than those with dry crop rotation (another dry crop as the preceding crop) for both datasets, while the difference in the recent dataset was significantly lower (*P*<0.01).

**Table 3:** Alien weed species, and their frequencies and change in dominance value (DV), among all sites surveyed between 1987–1992 (historical, 130 sites) and 2005–2010 (recent, 147 sites).

Species	Frequency recent (%)	Frequency historical (%)	Change in DV
*Geranium caroliniamum*	90.79	22.96	0.554*
*Conyza canadensis*	85.53	59.26	0.152*
*Alternanthera philoxeroides*	69.74	8.89	0.210*
*Vicia sativa*	65.79	65.93	−0.041 *^NS^*
*Veronica persica*	61.84	13.33	0.022 *^NS^*
*Erigeron annuus*	61.18	26.67	0.129*
*Avena fatua*	58.55	47.41	−0.029*
*Euphorbia helioscopia*	39.47	18.52	0.152*
*Sonchus asper*	28.95	0.74	0.014*
*Daucus carota*	26.97	4.44	0.044*
*Conyza bonariensis*	26.97	1.48	0.086*
*Bidens frondosa*	25.00	0	0.045*
*Conyza sumatrensis*	20.39	0.74	0.060*
*Aster subulatus*	14.47	1.48	0.011*
*Veronica polita*	12.50	34.81	−0.208*
*Coronopus didymus*	11.18	0	0.007 *^NS^*
*Cyperus rotundus*	11.18	5.19	0.005 *^NS^*
*Veronica arvensis*	7.24	17.04	0.019 *^NS^*
*Bidens pilosa*	4.61	0.74	0.014 *^NS^*
*Lepidium virginicum*	3.29	0	0.005 *^NS^*
*Aeschynomene indica*	3.29	0	0.003 *^NS^*
*Sonchus oleraceus*	3.29	1.48	0.004 *^NS^*
*Phytolacca americana*	3.29	0	0.004 *^NS^*
*Plantago virginica*	2.63	0	0.006 *^NS^*
*Bromus catharticus*	2.63	0	0.012 *^NS^*
*Ambrosia artemisiifolia*	2.63	0	0.003 *^NS^*
*Lolium temulentum*	1.97	7.41	−0.006 *^NS^*
*Thlaspi arvense*	1.97	8.89	−0.040 *^NS^*
*Amaranthus retroflexus*	1.97	0	0.000 *^NS^*
*Euphorbia maculata*	1.32	0	0.000 *^NS^*
*Crassocephalum crepidioides*	1.32	0.74	0.003 *^NS^*
*Lolium multiflorum*	0.66	0	0.002 *^NS^*
*Solidago canadensis*	0.66	0	0.001 *^NS^*
*Chenopodium ambrosioides*	0.66	0	0.000 *^NS^*
*Amaranthus tricolor*	0.66	0	0.000 *^NS^*
*Veronica hederaefolia*	0	2.96	−0.007 *^NS^*
*Coreopsis drummondii*	0	0.74	0.000 *^NS^*
*Veronica peregrina*	0	9.63	−0.005*

Note: “*”: *P*<0.05 and “*^NS^*”: not significant.

Note: Change in DV for each species in each grid was calculated by the DV in the recent dataset minus that in the corresponding historic dataset.

**Figure 4:**
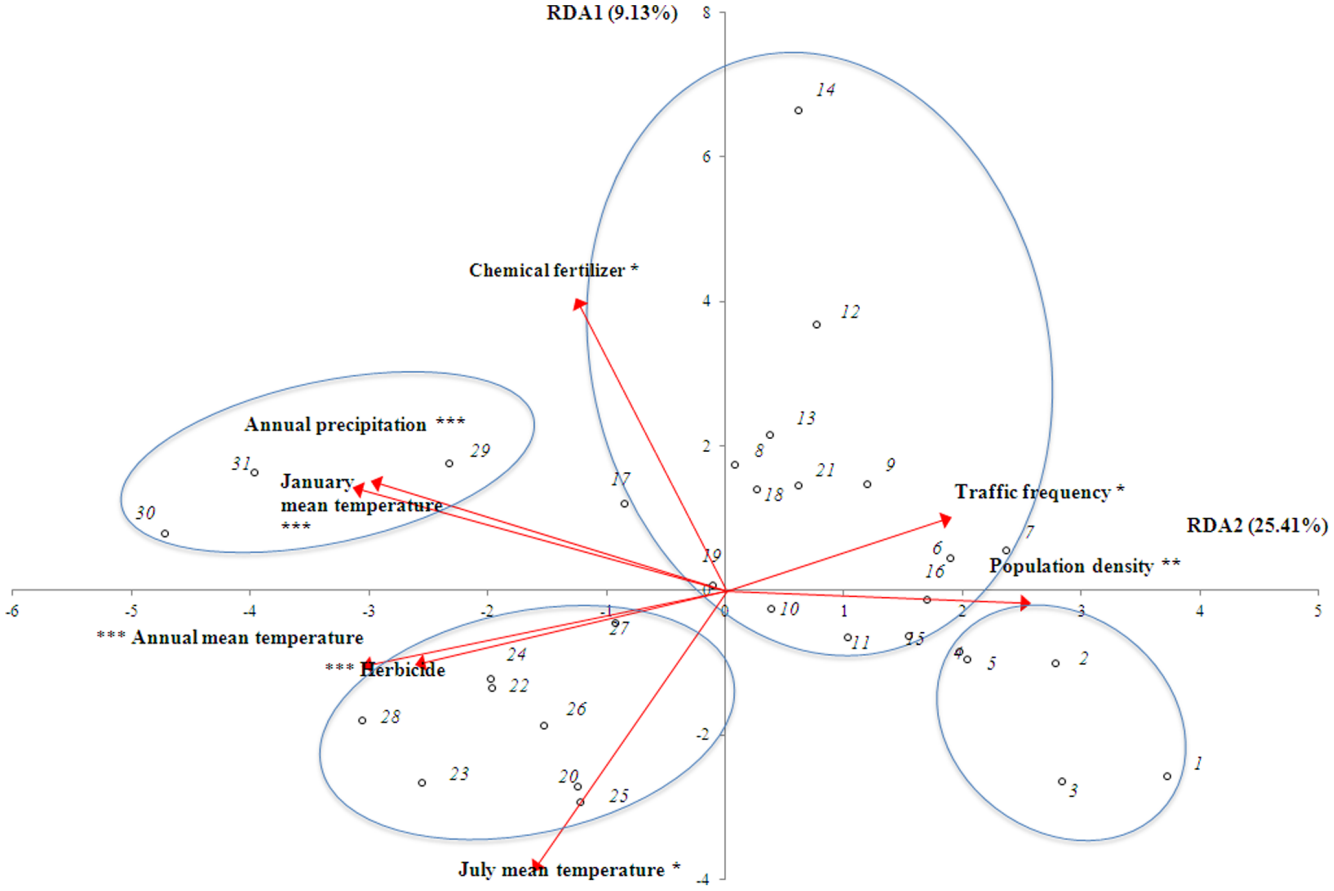
Redundancy analysis (RDA) showing the 8 significant environmental factors and the 31 geographic grids in Anhui Province, China (see Figure 1). RDA was conducted to analyze the relationship between changes in environmental factors and changes in the dominance values of alien weed species in croplands.

**Figure 5:**
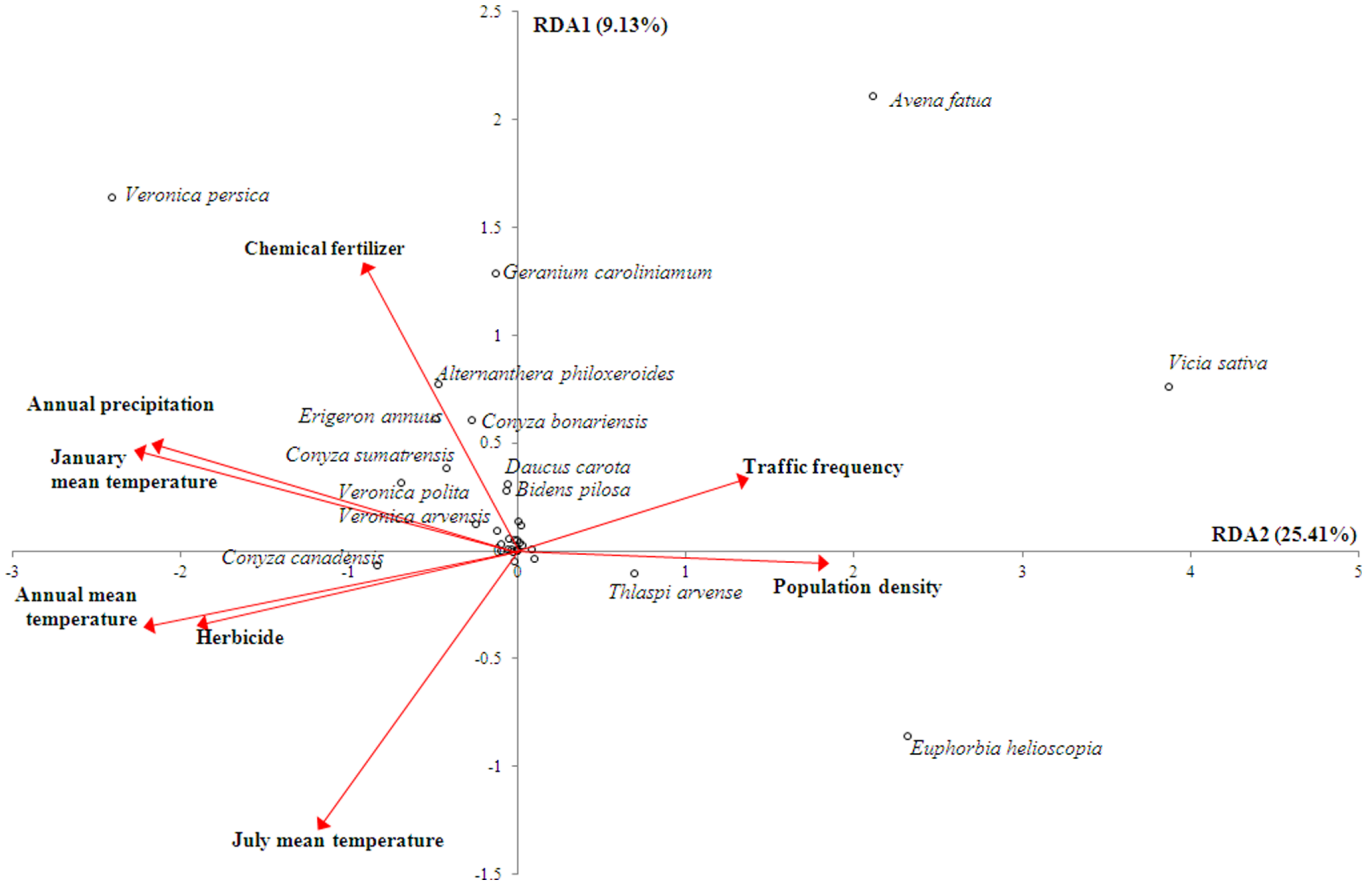
Redundancy analysis (RDA) showing the 8 significant environmental factors and alien weed species in croplands in Anhui Province, China. Note: species with lower correlations with RDA axes are not shown.

According to the RDA results, the 31 grids could be organized into 4 groups (Fig. 4), and increasing dominance of alien weed species was significantly related to changes in 8 environmental factors (Figs. 4 and 5). These factors included annual precipitation and mean temperature, mean temperature of the hottest (July) and coldest (January) month, amount of herbicide applied, amount of chemical fertilizer applied, population density, and traffic frequency. Among these8 factors, increases in population density and traffic frequency showed the greatest influences on changes in alien species invasions in north Anhui (grids 1 to 5); the dominance values of several alien weed species, including *Avena fatua* and *Euphorbia helioscopia*, increased significantly (*P*<0.05) with increased population density and traffic frequency(Fig. 5). Increased annual precipitation and mean January temperature facilitated invasion of alien weeds, including *Veronica persica*, *Alternanthera philoxeroides*, and *Conyza* spp., in south Anhui (grids 29 to 31).The increase in mean July temperature showed a negative influence on *A. fatua*, *V. sativa*, and *E. helioscopia*. A few species (e.g., *Veronica persica* and *Conyza canadensis*) benefited from increases in annual mean temperature and herbicide application, while species with higher invasiveness (*Geranium caroliniamum*, *A. fatua*, *V. specie*, *A. philoxeroides*, *Erigeron annuus*, *C. bonariensis*, and *C. sumatrensis*) benefited from increased fertilizer application.

Stepwise regression models revealed relationships between changes in alien weed richness and dominance and environmental factors. Six of the 8 environmental factors were significantly related to an increased richness of alien weeds (Table 4); the factor with the greatest influence was the increase in the amount of chemical fertilizer applied. The model identified 4 environmental factors that affected change in overall weed dominance, among which 3 (increased herbicide application, traffic frequency, and mean temperature of the hottest month) showed significant effects (Table 4). The greatest (positive) effect was due to the amount of herbicide applied.

**Table 4:** Results of the stepwise regression models used to test the relationships between changes in alien weed species richness and dominance, and changes in environmental factors between the 2 datasets surveyed in different time periods.

Parameter	Estimate	SE	*t*-value	*P*
**Alien weed species richness**				
Net cropland chemical fertilizerapplied	0.008	0.001	7.349	<0.001
Net cropland herbicide applied	−0.004	0.001	**−**5.660	<0.001
Population density	0.844	0.258	3.274	0.004
Net cropland agricultural machinery power	0.023	0.007	3.057	0.006
Annual mean temperature	−0.899	0.347	**−**2.592	0.017
Traffic frequency	0.950	0.448	2.119	0.046
Mean temperature of the coldestmonth	−0.180	0.089	**−**2.013	0.057
Mean temperature of the hottestmonth	0.048	0.030	1.606	0.123
ΔAIC with null model	−60.34			
**Overall dominance value of alien weeds**				
Net cropland herbicide applied	0.002	0.000	4.168	<0.001
Mean temperature of the hottestmonth	−0.137	0.054	**−**2.526	0.018
Traffic frequency	−0.446	0.188	**−**2.366	0.026
Mean temperature of the coldestmonth	0.328	0.169	1.948	0.063
ΔAIC with null model	−17.28			

The changes in Akaike’s information criteria between final and null models are also shown.

## Discussion

Plant invasion is a major economic problem in agricultural fields, and a variety of factors may influence the expansion of alien plants [24]. We collected data from field surveys that measured changes in diversity and dominance of alien agricultural weeds in relation to a historical survey. The most important factors identified as being correlated with increased diversity and dominance of alien weeds were the amount of chemical fertilizer and herbicide applied in agricultural areas. This finding has implications for the ways in which further plant invasions might be managed.

### Human Factors

Factors associated with human activities are frequently found to facilitate plant invasions [24]. Together, our results implied that increasing traffic frequency and population density, as well as changed farming practices, have significant influences on the occurrence of alien crop weeds.

Firstly, increased application of herbicides negatively affected the richness but positively affected the dominance of alien weeds in the surveyed area. Herbicide application can be an effective means of addressing invasion by the most serious weed species, and chemical weed control has greatly improved the efficiency of weed management in agricultural areas [14]. Many agricultural weed species are controlled simultaneously by herbicides, resulting in a decrease in species richness with increased herbicide application [25], as found in our study. However, many invasive plant species are capable of adapting to herbicides [1], such that chemical weed control may promote the dominance of invasive plants.

Many invasive plant species are also less sensitive to herbicides than are native plants or crop species. The active ingredients contained in the herbicides applied in our survey area primarily include 2, 4–D (2, 4-dicholrophenoxyacetic acid), MCPA (2-methyl-4-chlorophenoxy acetic acid), metsulfuron methyl, tribenuron-methyl, chlorsulfuron, isoproturon, fenoxaprop-P-ethyl, and quizalofop-p-ethyl [26]–[28]. *G. caroliniamum* showed higher tolerance than other species to most of the herbicides, [29], [30]. In the surveyed areas, *G. caroliniamum* increased in frequency from 23% to 91%, and its dominance value per site increased by 6 times from the historical to the current survey period. Similarly, *E. helioscopia* is reported to be insensitive to 2, 4–D, MCPA, and tribenuron-methyl [31], [32]; although increased herbicide application negatively impacted the dominance of this species, *E. helioscopia* maintained a high dominance value.

Long-term application of herbicides can result in selection and rapid distribution of herbicide-resistant weed species [33]. Our results suggested that the increased dominance of *C. canadensis* and *V. persica* was positively correlated with increased herbicide application (Fig. 5). Many invasive agricultural weeds show high genetic or phenotypic plasticity, and thus can become herbicide resistant in a short time, as has been reported for *C. canadensis* [34], *A. fatua* [35], *V. sativa* [36], and *Lolium multiflorum*. Ten years of intense glyphosate use (3.7 kg ha**^−^** ^1^ y**^−^** ^1^) in fruit orchards in Chile led to selection of glyphosate-resistant *L. multiflorum* populations [37]. The most-serious herbicide-resistant weed species in South America, such as *S. halepense*, *C. canadensis*, and *L. multiflorum*, are all invasive species [38]. Further, many serious herbicide-resistant weed species in North America, including *Ambrosia artemisiifolia*, *Ambrosia trifida*, *S. halepense*, *Amaranthus retroflexus*, and *C. canadensis* [33], [39] are serious invaders in countries outside of their native ranges [40]. Therefore, developing ecological weed management that is less reliant on herbicide application is of high importance [4], [41]; integrated agricultural practices such as rice-fish [42] and rice-duck [43] co-culture systems, and well-designed crop rotation and intercropping [44], could be part of the solution to this challenge.

The application of chemical fertilizer and use of agricultural machinery may promote alien plant invasions. Our results suggested that the increasing amount of chemical fertilizer applied was the most significant factor related to the increase of alien plant species richness (Table 4), and that increased quantities of chemical fertilizers applied to crops was positively related to increased dominance of several invasive plant species in the surveyed croplands. Combine harvesters can disperse weed seeds and other propagules over great distances, as shown for *A. fatua* [10] and *A. philoxeroides* (according to our field observation). Agricultural mechanization in Anhui Province is still at a low level, but is developing quickly [16]; hence, the potential influence of agricultural machinery on alien plant invasions deserves more attention.

In addition, our study suggested that alien plant invasions increased more rapidly in oilseed rape fields than in wheat fields. This could be the result of changes in the composition of alien plant species in the surveyed croplands. In the historical dataset, the most dominant alien species was *A. fatua*, followed by *V. sativa*, *Veronica polita,* and *V. persica*, all of which are archaeophytes (introduced before 1840).These 4 alien species account for 86% of total alien dominance value, and each of these species tended to occur in wheat fields. Nevertheless, several neophytes (introduced after 1840) in the surveyed areas spread quickly, particularly *G. caroliniamum*, *A. philoxeroides*, and *C. canadensis*, all of which tended to occur in oilseed rape fields. The continuous introduction and expansion of alien neophytes have resulted in plant invasions in oilseed rape areas becoming as serious as those in wheat fields. Other types of cropland that currently have lower rates of plant invasion may increasingly be faced with similar problems.

Alien plant invasions in lands with wet-dry crop rotation were less serious than those in lands with dry crop rotation, but increased faster. Alien invasions in areas with wet-dry crop rotation were very low in the historical dataset. One explanation may be that the annual shift in soil moisture between wet and dry planting periods caused a barrier against maintenance of weed seed banks [45], particularly for alien species that lack a long history of co-evolution with crop cultivation in China. Recently, some perennial alien plant species with rapid vegetative reproduction have invaded croplands and spread quickly, such as *A. philoxcroides* [46], which is highly adapted to different moisture conditions [47]. Seeds of some alien plant species, including *G. caroliniamum*, *V. sativa*, and *V. persica* [48] can remain viable in wet-dry crop rotation systems, and frequently infest this type of cropland. Moreover, some invasive weeds, such as *C. canadensis* and *E. annuus* [49], produce large amounts of small seeds and maintain large seed banks from which they readily disperse into croplands. Well-designed crop rotation systems could help to manage weed invasions. If possible, wet-dry crop rotation system showed higher resistance to alien plant invasions. Moreover, invasive weed species that are well adapted to both moist and dry soil should be more carefully monitored and controlled, and regular field investigations on crop weed communities should be conducted every few decades, as well the distribution of serious invasive plants should be monitored.

In addition, increases in human population density and traffic frequency both showed positive influences on the richness of alien plant species, consistent with studies that have shown correlations between anthropogenic disturbance and plant invasion [50]–[52]. Our results also suggested that the increased dominance of alien plants in areas with greater increases in traffic frequency was less pronounced in north Anhui Province. In north Anhui Province, several archaeophytes have been major agricultural weeds over long time periods, and the occurrence of these species did not increase significantly. For example, the most dominant alien weed species in the historical dataset were *A. fatua*, *V. sativa*, *V. persica*, *V. polita*, and *Thlaspi arvense*. Among these species, the dominance of *A. fatua*, *V. polita*, and *T. arvense* decreased significantly and those of *V. sativa* and *V. persica* did not change significantly.

### Physical Factors

Our results suggested that climatic change plays an important role in promoting the invasivity of alien plants in agricultural areas. Temperature and precipitation are widely known to be key factors in determining the distribution of plant species [3], [53], [54]. The increase in mean annual temperature and annual precipitation were positively correlated with increased dominance of several common invasive plants. However, the increase in mean temperature of the hottest month was negatively associated with dominance of most alien plant species. Located in the subtropical and temperate zones, Anhui Province has a climate with high precipitation, warm winters, and mild summers [16], which favor the establishment of a broad range of invasive plant species. Thus, the threat of plant invasions in this area may worsen in the future. The temperature in Anhui Province has shown a clear warming trend in recent decades [55], and annual precipitation has been highly variable with more frequent rainstorms [56]. A warming climate may enable the distribution of many invasive plants to expand [3]. Thus, cropping systems in many regions are likely to experience new vulnerabilities to exotic plant invasions in the future [11]. Additionally, more frequent rainstorms may result in increased flooding, which may further promote plant invasions by dispersing large numbers of seeds across a large area, as well as causing habitat fragmentation [57], [58].

### Conclusion

With increasing applications of herbicide and chemical fertilizer, higher population density, an upgraded traffic system, and the influence of climate change, plant invasions in crop areas have approximately doubled during the past few decades in Anhui Province, China. Differences in the seriousness of plant invasions among different types of cropping systems are fading. Much more attention should be focused on studying the current and potential distributions of invasive plants in agricultural areas, and to assess the risks of plant invasions in various croplands, particularly in areas that currently experience low rates of invasion. Considering that current patterns of biological invasion may better reflect historical than recent human activities [59], the potential for crop weed invasions in this area could be a serious threat in the next several decades and beyond. Integrative weed management with reduced application of chemical fertilizers and herbicides deserves much more attention.
